# Enhanced Gastric/Lung Arsenic Bioaccessibility from Lignite Fly Ashes: Comparing Bioaccessibility Rates with Multiple Environmental Matrices

**DOI:** 10.3390/toxics11040358

**Published:** 2023-04-10

**Authors:** Anna Bourliva, Efstratios Kelepertzis, Lamprini Papadopoulou, Carla Patinha, Nikolaos Kantiranis

**Affiliations:** 1 Directorate of Secondary Education of Western Thessaloniki, 56430 Thessaloniki, Greece; 2 Department of Geology and Geoenvironment, National and Kapodistrian University of Athens, Panepistimiopolis, Zographou, 15784 Athens, Greece; kelepert@geol.uoa.gr; 3 Department of Mineralogy-Petrology-Economic Geology, School of Geology, Aristotle University of Thessaloniki, 54124 Thessaloniki, Greece; lambrini@geo.auth.gr (L.P.); kantira@geo.auth.gr (N.K.); 4 GEOBIOTEC, Department of Geoscience, University of Aveiro, Campus de Santiago, 3810-193 Aveiro, Portugal; cpatinha@ua.pt

**Keywords:** lignite fly ash, arsenic, bioaccessibility, gastric, respiratory, mineralogy, Greece

## Abstract

Inorganic arsenic (As), a carcinogenic element to humans, is among the most dangerous and flammable substances that coal-burning plants could release. When coal is burned, large portions of arsenic are captured on fly-ash (FA) particles, but it could also contribute significantly to stack emissions of fine fly-ash particles. The aim of this study was to evaluate the oral and respiratory bioaccessibility of arsenic in lignite fly-ash (LFA) samples, and their contribution to total As exposure. Arsenic bioaccessibility fractions via ingestion and inhalation showed significant differences, suggesting the presence of highly soluble As-bearing phases in the studied LFA samples. The bioaccessible As fractions (BAF%) in the simulated gastric fluids (UBM protocol, ISO 17924:2018) showed a range of 45–73%, while the pulmonary bioaccessibility rates in the simulated lung fluid (artificial lung fluid (ALF)) exhibited significantly enhanced levels ranging from 86% to 95%. The obtained arsenic bioaccessibility rates were compared with previous data for multiple environmental matrices such as soil and dust-related materials, revealing that LFA exhibited significantly higher bioaccessibility (%) for the inhalation pathway.

## 1. Introduction

Arsenic (As), a Group I human carcinogen [1], is a ubiquitously available metalloid in Earth’s environment, typically occurring in +3 (arsenite) and +5 (arsenate) oxidation states in inorganic forms. The exposure to inorganic As through the consumption of contaminated food, water, and air, and occupational exposure have serious consequences for human health [2]. Arsenic enters the human body through a variety of routes, including ingestion, inhalation, and skin absorption. both pentavalent and trivalent arsenic compounds are rapidly and extensively absorbed from the gastrointestinal tract. Trivalent compounds are more water-soluble than pentavalent arsenic compounds and are thereby more toxic [3].

Arsenic exposure is a consequence of natural or anthropogenic sources. Adult and child exposure to As, specifically via drinking water, has resulted in a variety of complications, including dermatological, reproductive, neurological, and cardiovascular effects, and pulmonary disorders [3,4,5]. Furthermore, As ingestion may cause circulatory and neurological complications, and carcinogenesis in a variety of organs, including the skin, bladder, kidneys, lungs, and liver [6,7].

Arsenic is among the most dangerous and flammable substances that coal-burning plants can release [8]. The high elemental contents of As in fly ash are well-documented [9,10,11,12,13,14,15,16,17,18,19,20,21,22]. A sum of 23 European FAs sourced from coal-burning power plants exhibited As contents ranging from 22 to 162 mg kg^−1^ with an average of 55 mg kg^−1^ [17]. Regarding Greek fly ash, Georgakopoulos et al. [10] reported As contents ranging from 20.5 to 38.8 mg kg^−1^, while Samara [8] found As contents up to 46.9 mg kg^−1^. Moreover, the chemical speciation of As in FA is essential and was extensively studied [23,24,25]. Arsenic in FAs occurs mainly as pentavalent arsenate As(V) with minor trivalent arsenite As(III), which might be associated with glass, iron (oxyhydr)oxides, and calcium arsenate.

Arsenic, in contrast to other elements, shows strong affinity to the finest fly-ash fractions [26,27]. Fly ash with smaller particles could readily pass through the body’s respiratory system, enter the stomach, and float in the air. Thus, incidental ingestion and inhalation are important non-dietary As exposure pathways for both adults and children [28]. Although exposure assessment was previously based on total metal(loid) content, bioavailability (i.e., absorption in the systemic circulation) and/or bioaccessibility (i.e., solubility in simulated biological fluids)-based approach is currently dominating that offers more accurate exposure quantification. The limitations and ethical considerations of *in vivo* exposure assessment methods have led to in vitro bioaccessibility methods being a potential alternative that supplements data for exposure assessment [29]. As a result, information is critical on bioaccessible As fractions, i.e., soluble As fractions in the target compartment’s conditions (gastrointestinal or respiratory).

The high toxicity of As, especially if one is exposed via inhalation [30,31], indicates the importance to investigate its bioaccessibility to better quantify exposure. A growing number of studies were conducted in this context to determine the bioaccessible fraction of As in particulate matter, soil, and dust [32,33,34,35,36,37]. Despite significant concerns about the health risks of As release in simulated biological fluids, there are relatively few studies on gastric/lung As bioaccessibility from fly ash. Jin et al. [38] reported that bioaccessible (gastric and intestinal) arsenic was in the range of 36–67% for Chinese fly-ash samples. Lokeshappa et al. [39] determined that more than 40% of As was in bioaccessible form via ingestion from Ca-rich and Si-rich fly ash using a physiological extraction test (PBET). The objectives of the present study are to (a) evaluate As distribution in ingestible and inhalable size fractions of Greek lignite fly ash (LFA), (b) determine bioaccessible As fractions via the ingestion and inhalation of LFA, and (c) compare the bioaccessibility rates with published data.

## 2. Materials and Methods

### 2.1. Lignite Fly-Ash Samples

We used five lignite fly-ash (LFA) samples (AD, KD, AM, MG1, MG2) produced in lignite-fired power plants operating in the main lignite basins of Greece. Details of the power plants (PPs), sample pretreatment, and the main physicochemical characteristics were presented elsewhere [40,41]. We studied Ca-rich LFA from plants operating in the Ptolemais–Amynteo (P–A) lignite basin (AD, Agios Dimitrios PP; KD, Kardia PP; AM, Amynteo PP) and Si-rich LFA from power plants operating in the Megalopolis basin (MG1 and MG2). Ingestible (<250 μm) and inhalable (<10 μm) particle size fractions were separated with dry sieving for the gastric and lung bioaccessibility tests, respectively. All samples were sealed and stored (<4 °C) until further analysis.

### 2.2. Analytical Methods

The mineralogical characterization of the studied LFA size fractions (<250 and <10 μm) was performed with a water-cooled Rigaku Ultima diffractometer with CuKa radiation, a step size of 0.05°, and a step time of 3 s operating at 40 kV and 30 mA. Morphological characteristics and differences among LFA particle sizes were examined with scanning electron microscopy (SEM) on a JEOL JSM-840A microscope operating at 20 kV connected with an X-ray energy dispersive spectrometer (EDX; INCA 300).

The elemental concentrations of major elements Si, Ti, Al, Fe, Mn, Mg, Ca, K, P, and S were determined via pXRF. A Bruker S1 Titan 600 with a 4 W Rh X-ray tube, 5 mm spot size, a silicon drift detector (resolution < 145 eV), and the inbuilt Geoexploration mode, a factory calibration method for soils, was used for pXRF analysis. The analysis was conducted in three energy ranges (15, 30 and 50 kV) for 30 s each (a total of 90 s). The elemental contents calculated with the Geoexploration mode were calibrated using certified reference materials (CRMs); calibration curves with R^2^ values higher than 0.9 were obtained.

The near-total As contents were determined after an aqua regia digestion using inductively coupled plasma mass spectrometry (ICP–MS) at ActLabs Analytical Laboratories Ltd., Canada (for the ingestible size fraction < 250 μm) and GeoBioTec Laboratory, University of Aveiro, Portugal (for the inhalable size fraction < 10 μm). Quality assurance and quality control (QA/QC) included reagent blanks, analytical replicates, sample duplicates, and analyses of inhouse reference materials. The recovery rates were estimated within ±10% of the certified value, and analytical precision (expressed as RSD %) was <10%.

Both the oral and lung bioaccessibility determinations of As were carried out in GeoBioTec, University of Aveiro, Portugal. The <250 μm particle size was utilized for the oral bioaccessibility tests since this soil portion adheres to human hands and becomes ingested during hand-to-mouth activity [42]. Smaller particles, specifically the <10 μm size fraction, were selected for the lung bioaccessibility tests since they could be deposited in the tracheal–bronchial and alveolar region, thus posing the greatest risk via inhalation [29].

Oral bioaccessibility was determined using the unified bioaccessibility method (UBM) validated against an in vivo model [43]. The UBM protocol [44] is a two-stage in vitro procedure that simulates the leaching of a solid matrix in both the gastric (G) and gastrointestinal-tract (GI) phases by using synthetic digestive solutions according to physiological transit times. Specifically, the procedure consists of consecutive steps in simulated human saliva and gastric juice (G phase, 37 °C, 1 h, pH 1.2 ± 0.05), and then in simulated bile and duodenal fluid (GI phase, 37 °C, 4 h, pH 6.3 ± 0.5). The samples were shaken at 37 °C on an end-over-end shaker, and the particles were separated from the solutions with centrifugation at 4500× *g* for 15 min. Lung bioaccessibility determinations were performed using an artificial lysosomal fluid (ALF) solution (pH 4.5) that simulates intracellular conditions in the lungs and extracts higher concentrations of metals than those of other commonly used simulated lung fluids [45]. In brief, 0.05 g (±0.0001) of the inhaled size fraction (<10 μm) was weighed into 85 mL polycarbonate centrifuge tubes, and 50 mL of the simulated fluid (ratio of 1:1000) was added. The samples were shaken at 37 °C on an end-over-end shaker for 24 h, and the particles were separated from the solution by centrifugation at 4500× *g* for 15 min. Both digestive and lung simulated biological fluids (SBFs) were freshly prepared prior to extraction. Bioaccessible oral and pulmonary As (including both As^3+^ and As^5+^) concentrations (mg kg^−1^) in the supernatants were prepared with ICP–MS. UBM-specific certified reference soil BGS102 (ironstone soil from Lincolnshire, UK) that provides UBM guidance values [46,47] was used in order to validate the uncertainty of the extraction protocol. For BGS102, the recovery rate for As was 115.4% for the gastric phase and 113.2% for the gastrointestinal phase. Though respiratory bioaccessibility values for several elements were reported for standard reference material BCR-723 [45], there are no reported values for arsenic.

## 3. Results and Discussion

### 3.1. Mineralogy and Morphology of LFA Size Fractions

**Figure 1:**
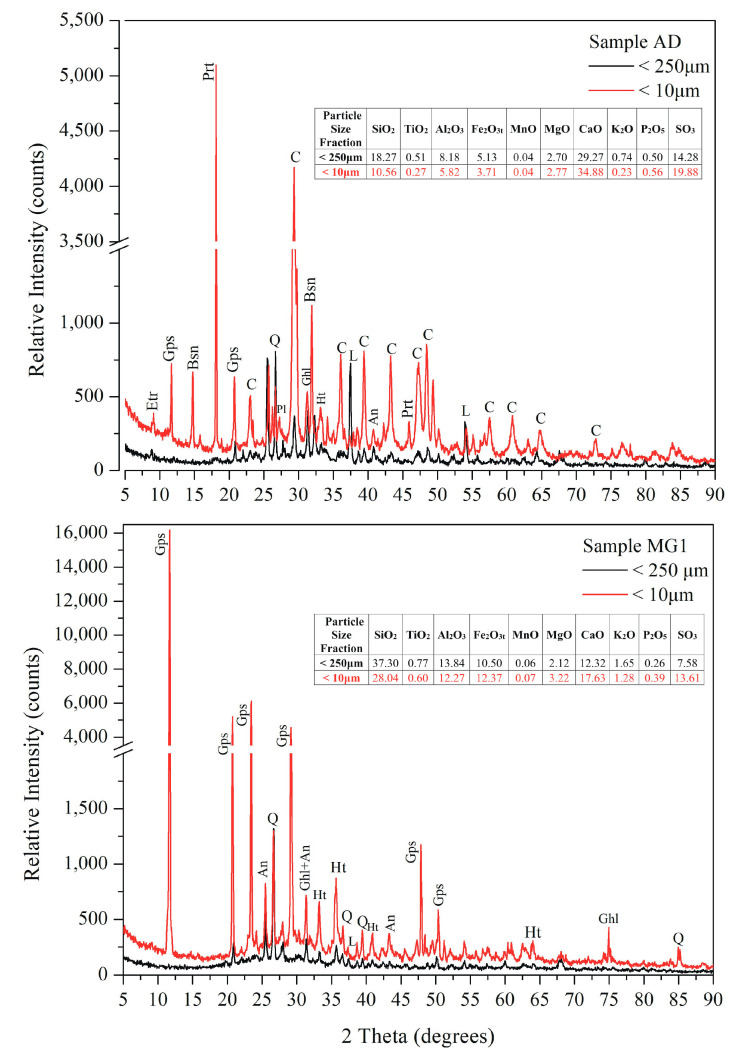
Representative XRD patterns of Ca-rich (Sample AD, **top**) and Si-rich (Sample MG1, **bottom**) fly-ash samples of the two different studied size fractions. An: anhydrite, Bsn: bassanite, C: calcite, Etr: ettringite, Ghl: gehlenite, Gps: gypsum, Ht: hematite, L: lime, Prt: portlandite, Pl: plagioclase, Q: quartz. The concentration units of pXRF results are given in the inlets.

The mineralogical composition of the bulk (<250 μm) LFA samples was discussed elsewhere [41]. Here, the mineralogical differences in the different particle sizes were further investigated, and representative XRD patterns are given in Figure 1.

As reported [41], ingestible LFA fractions (<250 μm) contained both amorphous and crystalline mineral phases with quartz and a number of Ca-bearing mineral phases dominating. Significant differences in the mineralogy of the inhalable size fraction (<10 μm) of Ca- and Si-rich LFA were revealed. Specifically, quartz content decreased with the decrease in particle size, indicating that quartz tended to aggregate in the coarse fly-ash fractions. Inversely, the calcium mineral content greatly increased with the decrease in particle size, showing a relatively high content of Ca-bearing minerals in finer LFA fractions. Specifically, Ca-rich LFA revealed high contents of secondary Ca-bearing mineral phases such as calcite (CaCO_3_), Ca hydroxides (i.e., portlandite) and Ca sulphates (i.e., gypsum, bassanite) and/or Ca–Al sulphates (i.e., ettringite). Similarly, Si-rich LFA exhibited significantly higher Ca-bearing phases (i.e., gypsum, calcite), in contrast with the coarser fractions, where Si-rich minerals (i.e., quartz, feldspars) dominated. Moreover, Fe-bearing phases (i.e., hematite) tended to accumulate in the finer LFA fractions. The aforementioned findings are supported by XRF analysis in both size fractions (Figure 1, inlets).

**Figure 2:**
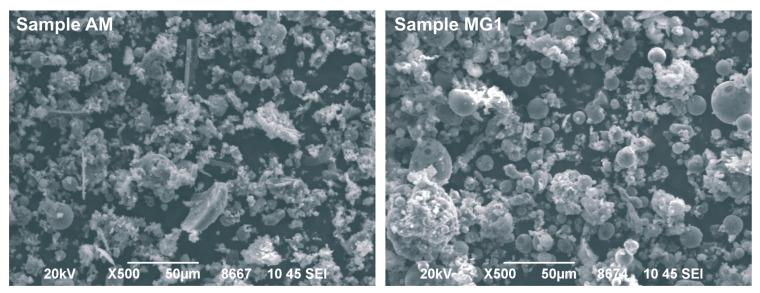
Representative SEM images of the studied fly-ash samples.

SEM observations revealed the dominance of typical spherular fly-ash particles with sizes ranging from 1 to 100 μm, while irregular aggregates and needlelike particles (mainly Ca sulphates) were also observed (Figure 2). As already reported [41], Ca-rich LFA were dominated by irregularly shaped Si-rich particles and angular Ca-rich agglomerates in a wide range of sizes, while Si-rich LFA exhibited a plethora of Si-rich and/or Fe-rich spherules with lower contents of Ca, Al, and Mg.

### 3.2. Arsenic Contents in LFA Size Fractions

**Figure 3:**
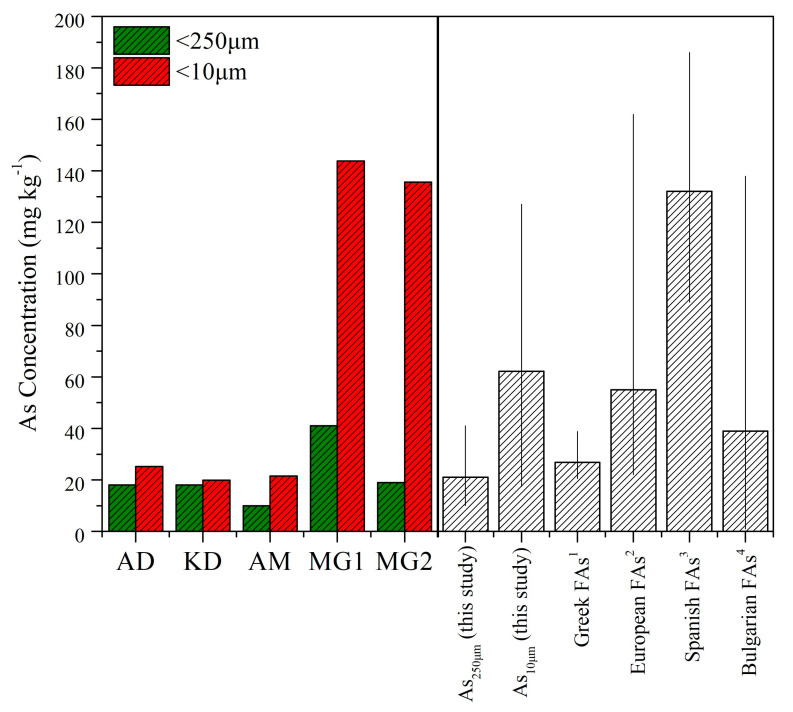
Arsenic concentrations (mg kg^−1^) in the <250 and <10 μm size fractions, and a comparison of the obtained As contents with published data ^1^ [10], ^2^ [17], ^3^ [49], ^4^ [48].

Near-total As concentrations in the studied LFA size fractions are illustrated in Figure 3. Near-total As concentrations in the <250 μm size fraction (total As_250 μm_) ranged between 10 and 41 mg kg^−1^ with a mean As content of 21.2 mg kg^−1^ (Figure 3). The concentration of As in the finer fraction (total As_10 μm_) exhibited a mean value of 69.2 mg kg^−1^ with severe variation in As content among the LFA samples from different power plants. Specifically, total As_10 μm_ varied from 19.8 (KD) to 143.9 mg kg^−1^ (MG1). The descending order of MG1 > MG2 > AD > AM > KD was observed, with significantly higher As contents being recorded in the Si-rich LFA (MG1 and MG2). Specifically, Si-rich LFA exhibited an average of almost 140 mg kg^−1^ of As compared to an average of 22 mg kg^−1^ in Ca-rich LFA. Moreover, a negative correlation was revealed between As concentration and particle size. As contents were increased with the decrease in particle size from 250 to 10 μm; especially for Si-rich LFA, this increase was quite sharp. Specifically, total As_10 μm_ was almost 3 to 7 times higher than the corresponding one in the coarser fraction (<250 μm) for MG LFA samples, and up to 2.2 times higher for the P-A LFA samples.

The findings demonstrate As enrichment in finer LFA particles and are in agreement with earlier reports [11,38]. Although a direct comparison of As contents is difficult due to the different applied analytical methods and utilized particle size fractions, the obtained As concentrations in the <250 μm size fraction were relatively lower than those reported for European coal fly ash [17] and globally for different FA types [48,49,50]. However, the finer LFA fractions were significantly enriched in As, with the average within the reported ranges of arsenic for European FA (Figure 3).

### 3.3. Arsenic Gastric/Lung Bioaccessibility Data

**Figure 4:**
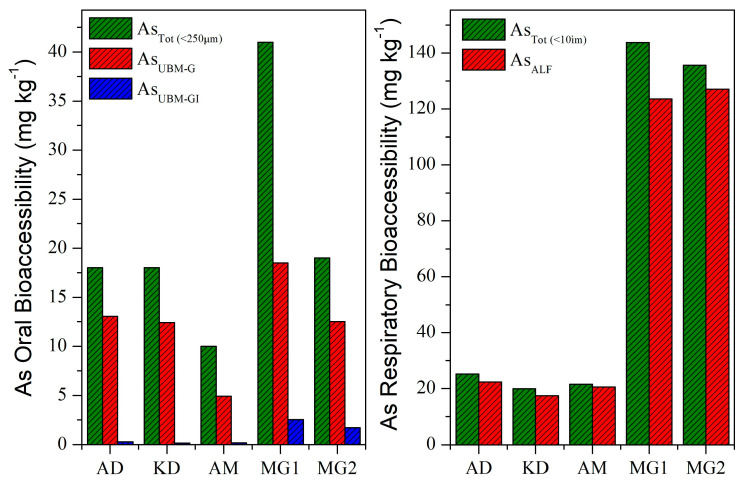
Arsenic bioaccessibility (in mg kg^−1^) of lignite fly-ash samples in (**left**) gastric (G)/gastrointestinal (GI) and (**right**) lung fluids.

The bioaccessible As concentrations (mg kg^−1^) in the simulated gastric/gastrointestinal and lung fluids are presented in Figure 4.

The UBM-extractable As contents in the gastric phase ranged from 4.9 (AM) and 18.5 mg kg^−1^ (MG1) with an average value of 12.3 mg kg^−1^. The UBM-extractable As concentrations (G-phase) in the studied LFA exhibited the decreasing order of MG1 > AD > MG2 > KD > AM, with a significant correlation between UBM-extractable As concentrations and total As contents (r^2^ = 0.897). Significantly lower UBM-extractable As contents were recorded in the GI phase with the range of 0.2–2.5 mg kg^−1^. Such a decrease in As bioaccessible contents in the simulated intestinal phase was also reported by Jin et al. [38]. These surprisingly lower bioaccessibility values are indicative of the influence of the matrix type that, in combination with parameters in the simulated GI-phase, may lead to the complexation and precipitation of As from the solution, and needs further investigation. On the other hand, ALF-extractable As contents (mean, 62.2 mg kg^−1^) were higher than the corresponding UBM-extractable ones (mean, 12.3 mg kg^−1^) in the gastric phase, especially for Si-rich LFA samples (Samples MG1 and MG2). The higher extractable contents in the lung fluid (ALF) indicate the aggressiveness of the method [45]. ALF-extractable As contents exhibited great variability among samples, with values ranging from 17.5 (KD) to 127.1 mg kg^−1^ (MG2); a different descending order was recorded: MG2 > MG1 > AD > AM > KD. A strong correlation (r^2^ = 0.998) between total and ALF-extractable As contents suggests that total As contents might be a good indicator of lung bioaccessibility.

**Figure 5:**
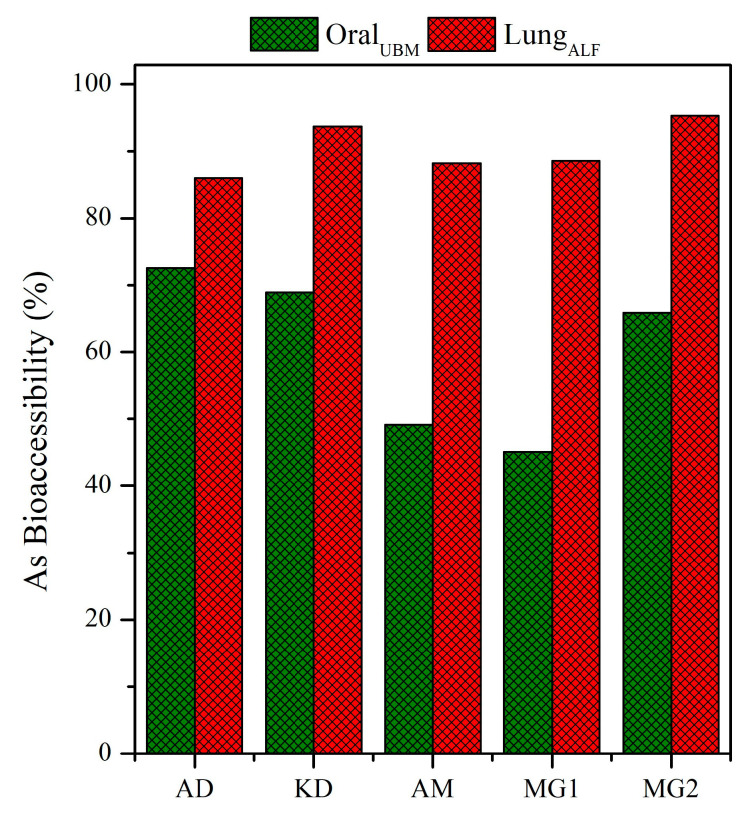
Arsenic bioaccessibility (%) of lignite fly-ash samples in gastric and lung fluids.

A different trend on gastric/lung bioaccessibility was revealed when extractable As contents were expressed as percentages with respect to the total As contents (Figure 5). The bioaccessible As fractions (BAF%) via ingestion showed the range of 45.1–72.5%. The descending order of AD > KD > MG2 > AM > MG1 was recorded, indicating that As was relatively more bioaccessible in the Ca-rich LFA, which was in line with our previous study concerning multiple trace elements [41]. Significantly higher bioaccessible As fractions were recorded in the artificial lung fluid ranging between 86% (AD) and 95% (MG2). Despite the differences in total As_10 μm_ contents from the five plants, the bioaccessible As fractions via inhalation were similar, suggesting the presence of highly soluble As-bearing phases in all samples.

### 3.4. Correlation between As Bioaccessibility and Elemental Contents

**Figure 6:**
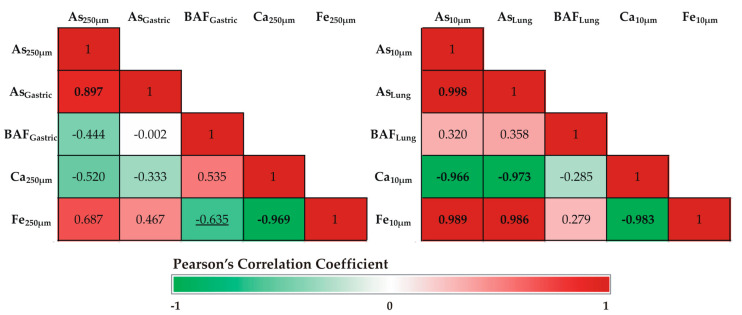
Heat maps of Pearson’s correlation coefficients matrix for (**left**) gastric and (**right**) lung As bioaccessibility and selected geochemical parameters. Correlations in red are positive and in green are negative. *p* < 0.01 significance is marked in bold, and *p* < 0.05 in underlined.

Pearson correlation coefficient was used to investigate the relationship between As bioaccessibility and selected geochemical parameters (Figure 6).

Gastric As was strongly correlated with total As_250 μm_, indicating that, as the total concentrations of As increased, the bioaccessible As in the gastric fluids also increased. Bioaccessible As via ingestion did not show any significant correlations to any other element. However, the gastric bioaccessible fractions of As were significantly negatively correlated with total Fe_250 μm_ (r^2^ = −0.635), indicating a strong inverse relation of As bioaccessibility to Fe-bearing phases. A difference in As bioaccessibility to Fe-rich phases was reported indicating a decrease in the As bioaccessible fraction with an increase in both the number of Fe-rich phases and their crystallinity [51,52,53].

Similarly, lung As was strongly correlated with total As_10 μm_, while strong correlations were also revealed for total Fe_10 μm_ (r^2^ = 0.989) and Ca_10 μm_ (r^2^ = −0.966). In contrast, no correlations were recorded for bioaccessible As fractions via inhalation and elemental contents.

### 3.5. Comparison of Bioaccessible Rates with Multiple Environmental Media

**Figure 7:**
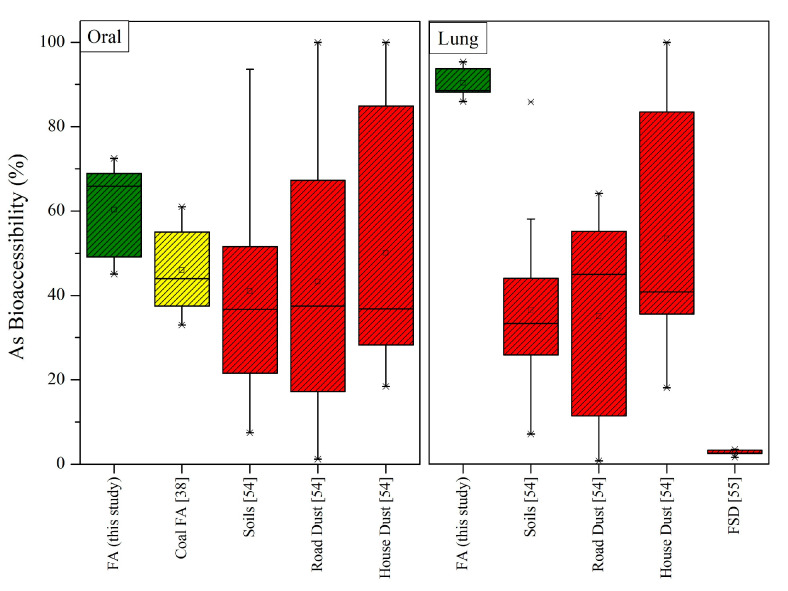
Comparison of As bioaccessible fractions (%) between LFA and other environmental materials. Data for other coal FAs are from Jin et al. [38], for soil, road dust, and house dust are from Kelepertzis et al. [54], and those for fine surface dust (FSD) are from Morais et al. [55]. The box is determined by the 25^th^ and 75^th^ percentiles. The whiskers (−) are determined by the 5^th^ and 95^th^ percentiles. Additionally, the minimum (*), median (line), mean (□) and maximum (*) values are presented. Green boxes: this study; Yellow boxes: other FAs; Red boxes: other materials.

The literature shows variability in As bioaccessibility in simulated biological fluids. Considering the differences in particle size fractions and the applied in vitro arsenic dissolution protocols, direct comparisons of the bioaccessibility rates among published studies should be treated with caution. Here, we utilized bibliographic data considering As bioaccessibility from multiple environmental media, including coal fly ash [38], soil, and road and house dust from a heavy industrial area [54], and dust from a gold-mining district [55] for comparisons, and the results are illustrated in Figure 7.

Jin et al. [38] estimated oral As bioaccessibility for FA from coal-fired power plants from North China. According to their report, the proportion of bioaccessible (gastric and intestinal) As accounting for total As was in the 36–67% range, which are significantly lower rates than those obtained in our study (Figure 7). According to Kelepertzis et al. [54], the median oral As bioaccessible fractions were 35% for soil, 38% for road dust, and 35% for house dust, while the bioaccessible fractions for the inhalation pathway were 33% for soil, 45% for road dust, and 40% for house dust. Lastly, gastrointestinal and lung bioaccessibility tests on the fine surface dust (FSD) from a gold-mining district [55] yielded surprisingly lower bioaccessible concentrations of As in the surface soil (3.4 ± 2%) and residential FSD samples (2.7 ± 1%).

The above indicate that both gastric and lung bioaccessible As fractions (%) showed great variation among the compared matrices. Specifically, for the ingestion pathway, As bioaccessibility followed the order of LFA > house dust ≥ road dust > soil; for the inhalation pathway, the order was similar, namely, LFA > house dust > soil ≥ road dust > FSD, suggesting the presence of highly soluble As-bearing phases in the studied LFA samples. Interestingly, the differences in BAF (%) between the LFAs and the other environmental media were more pronounced for respiratory bioaccessibility. The overall comparison of the BAFs of As in the studied LFAs with data from several authors for a variety of environmental sampling media clearly demonstrate the significantly higher As bioaccessibility in LFA, especially for the inhalation pathway. of the sources of variability are probably related to the physicochemical factors of the samples (sample type, particle size, speciation) or even the protocol parameters (extraction time, solid/liquid ratio, temperature, and agitation). For instance, the inhalation bioaccessibility results on the applied SLF presented by Kelepertzis et al. [54] were on a coarser size fraction (<100 μm) for which the rate and extent of As dissolution was probably diminished; consequently, lower As bioaccessibility results were potentially obtained. Moreover, variation in the bioaccessibility rates indicates that the simulated lung fluids “attacked” either different As-containing phases or identical phases with varying intensity. The importance of metal(loid) speciation in influencing bioaccessibility was also extensively reported [51,53].

## 4. Conclusions

In this study, As contents and bioaccessible fractions in ingestible (<250 μm) and inhalable (<10 μm) size fractions of Greek lignite fly ash (LFA) were evaluated. Lignite fly-ash samples of different chemical types (i.e., Ca-rich and Si-rich) were from coal-fired power plants operating in the main lignite basins of Greece. Differences in the mineralogical composition of the studied size fractions were revealed. Specifically, quartz exhibited increased contents in the coarser LFA fractions (<250 μm), while Ca-bearing mineral phases were relatively enriched in the finer LFA fractions. Arsenic contents in the ingestible size fraction (total As_250 μm_) exhibited the range of 10–41 mg kg^−1^, while significantly higher concentrations were recorded in the inhalable LFA fraction (total As_10 μm_) ranging from 19.8 mg kg^−1^ (Ca-rich LFA sample) to 143.9 mg kg^−1^ (Si-rich LFA sample), indicating an As enrichment character in fine fractions that should raise public concern. Gastric As bioaccessible fractions ranged from 45.1% to 72.5%, while 86–95% of As was bioaccessible via the inhalation pathway. Though the comparison of bioaccessibility rates should be treated with caution, Greek lignite fly ash demonstrates substantial higher bioaccessibility rates, especially via the inhalation pathway, indicating that the investigated LFA samples contained highly soluble As-bearing phases, which is highly concerning. These findings highlight the significance of characterizing the bioaccessibility rates of LFA in health risk assessment, and this may help in guiding ongoing efforts to reduce public health risk.

## Data Availability

The presented data in this study are contained within the article.
